# Factors associated with development of symptomatic disease in Ethiopian COVID-19 patients: a case-control study

**DOI:** 10.1186/s12879-021-06465-1

**Published:** 2021-08-05

**Authors:** Tigist W. Leulseged, Degu G. Alemahu, Ishmael S. Hassen, Endalkachew H. Maru, Wuletaw C. Zewde, Negat W. Chamiso, Kalkidan T. Yegele, Daniel S. Abebe, Firaol M. Abdi, Etsegenet Y. Minyelshewa, Tegenu G. Gerbi, Helen T. Hagos

**Affiliations:** Department of Internal Medicine, Research Development Office, Millennium COVID-19 Care Center, St. Paul’s Hospital Millennium Medical College, Addis Ababa, Ethiopia

**Keywords:** Symptomatic/ asymptomatic COVID-19, Case-control, Logistic regression, Ethiopia

## Abstract

**Background:**

Studies show that having some symptoms seems to be associated with more severe disease and poor prognosis. Therefore, knowing who is more susceptible to symptomatic COVID-19 disease is important to provide targeted preventive and management practice. The aim of the study was to assess factors associated with the development of symptomatic disease among COVID-19 patients admitted to Millennium COVID-19 Care Center in Ethiopia.

**Methods:**

A case-control study was conducted from August to September 2020 among a randomly selected 730 COVID-19 patients (337 Asymptomatic and 393 Symptomatic patients). Chi-square test and independent t-test were used to detect the presence of a statistically significant difference in the characteristics of the cases (symptomatic) and controls (asymptomatic), where *p*-value of < 0.05 considered as having a statistically significant difference. Multivariable binary logistic regression was used to assess a statistically significant association between the independent variables and developing symptomatic COVID-19 where Adjusted Odds ratio (AOR), 95% CIs for AOR, and *P*-values were used for testing significance and interpretation of results.

**Results:**

The result of the multivariable binary logistic regression shows that age group (AOR = 1.89, 95% CI = 1.25, 2.87, *p*-value = 0.002 for 30–39 years; AOR = 1.69, 95% CI = 1.06, 2.73, *p*-value = 0.028 for 40–49 years and AOR = 4.42, 95% CI = 2.75, 7.12, *p*-value = 0.0001 for ≥50 years), sex (AOR = 1.76, 95% CI = 1.26, 2.45, *p*-value = 0.001) and history of diabetes mellitus (AOR = 3.90, 95% CI = 1.92, 7.94, *p*-value = 0.0001) were found to be significant factors that determine the development of symptomatic disease in COVID-19 patients.

**Conclusions:**

Developing a symptomatic COVID-19 disease was found to be associated with exposures of old age, male sex, and being diabetic. Therefore, patients with the above factors should be given enough attention in the prevention and management process, including inpatient management, to pick symptoms earlier and to manage accordingly so that these patients can have a favorable treatment outcome.

**Supplementary Information:**

The online version contains supplementary material available at 10.1186/s12879-021-06465-1.

## Background

The new coronavirus disease 2019 (COVID-19) caused by a virus in the coronaviruses family called severe acute respiratory syndrome coronavirus 2 (SARS-CoV-2) was first identified in China in December 2019 and declared to be a pandemic by the World Health Organization in March 2020 [1].

Till today, there is no such pathognomonic feature identified for the disease presentation. But according to studies, the COVID-19 disease could present with different symptoms and signs. Regarding the spectrum of symptoms, studies show that the presentation spectrum could range from having no symptoms at all while being infected (asymptomatic cases) to different types of one or more symptoms. Respiratory and constitutional symptoms are reported to be the commonest presentation in most setups [2–4].

In addition, symptoms like hyposmia, hypogeusia, nasal congestion, rhinorrhea, sputum, cerebrovascular accidents, abdominal pain, vomiting, and diarrhea are reported with varying frequency from place to place [2–8].

Studies about what determines developing symptomatic disease are lacking. But different studies and reports point that acquiring the disease, presenting with symptoms, having severe disease and bad prognosis shows a difference in terms of sex showing that the male sex is more vulnerable to worse conditions. Different explanations are given starting from the genetic level up to behavioral factors that could cause men to be more susceptible to the disease [9–15]. Studies including the ones conducted in our center showed that having some symptoms seems to be associated with more severe disease and poor prognosis rather than other symptoms or not having symptoms at all. Therefore, knowing who is more susceptible to developing symptomatic COVID-19 disease is important to provide a better preventive practice and stratified patient management and follow up. Therefore, the objective of this study was to assess factors associated with the development of symptomatic disease among COVID-19 patients admitted to Millennium COVID-19 Care Center in Ethiopia.

## Methods

### Study design and setting

An institution based case-control study was conducted at biggest make shift COVID-19 Center, Millennium COVID-19 Care Center (MCCC), in Addis Ababa, Ethiopia with referral in from all over the country. The center started work on June 2, 2020 with a capacity of 1000 beds including 40 ICU beds. Due to the late detection of the virus in the country and the immediate lock down, all individuals with a positive RT-PCR result gets admitted to the center irrespective of the presence or absence of symptom, severity of the disease, age and other baseline medical conditions, with the intention of containing the infection and preventing further spread. Asymptomatic patients were identified and admitted through the contact tracing system of the Ethiopian Public Health Institute. At the time of this study, patients were discharged with viral clearance criteria as evidenced by two negative RT-PCR tests done at least 24 h apart but eventually with the change in admission pattern, the discharge criteria also changed to clinical improvement (resolution of signs and symptoms). At the time of the study, the Centre was one of the few COVID-19 centres in the country with a large flow of patients from all over the country.

### Source and study population

The source population was all patients admitted to MCCC with a confirmed diagnosis of COVID-19 using RT-PCR by a laboratory given mandate to test such patients by the Ethiopian Federal Ministry of Health [16].

- Case (symptomatic): all RT-PCR confirmed COVID-19 patients admitted to the MCCC and who presented with one or more symptoms at admission and during the clinical course of the disease.
- Control (asymptomatic): all RT-PCR confirmed COVID-19 patients admitted to the MCCC and who did not have any symptoms during the clinical course of the disease.

The study population was all selected COVID-19 patients who were on treatment and follow up at MCCC and who full fill the inclusion criteria.

### Sample size determination and sampling technique

The sample size was determined using sample size calculation for a case-control study with the following assumptions; 95% confidence interval, power of 90%, one to one ratio of cases and controls, the proportion of males who are symptomatic as 0.85, and proportion of females who are asymptomatic as 0.75 and considering a non-response rate of 10%. Therefore, the total sample size calculated becomes 778 cases of COVID-19 (389 symptomatic and 389 asymptomatic).

To select both the cases and the controls, simple random sampling method using table of random numbers was used.

### Eligibility criteria

All COVID-19 patients who were on treatment and follow up at MCCC and whose clinical history regarding presenting symptom and the relevant explanatory variables is well documented were included.

### Operational definition

#### Asymptomatic patient

Any patient who has tested positive for COVID-19 but does not have any subjective symptoms. These patients are detected after isolation and contact tracing as done by Ethiopian Public Health Institute [16].

### Data collection procedures and quality assurance

A data abstraction tool to pick all the relevant variables was drafted based on the patient registration and follow-up form and then pretested on 5% of randomly selected charts which were not included in the final data collection. The final data abstraction tool was then developed after modification based on the pretest. Data was collected by six trained General practitioners.

Data consistency and completeness were checked before an attempt was made to enter the code and analyze the data.

### Data management and analysis

The extracted data were coded, entered into Epi-Info version 7.2.1.0, cleaned, stored, and exported to SPSS version 25.0 software for analysis. Categorical covariates were summarized using frequencies and percentages and numerical variables were summarized with a mean value (± Standard deviation). A chi-square test and an independent t-test were run to determine the presence of a significant difference between the independent variables and the development of symptom. A statistically significant difference was detected for variables with a *P*-value of ≤0.05.

The association between the dependent variable and independent variables was analyzed using multivariable Binary Logistic Regression. Univariate analysis was done at a 25% level of significance to screen out independent variables used in the multiple Binary Logistic regression model. The adequacy of the final model was assessed using the Hosmer and Lemeshow goodness of fit test and the final model fitted the data well (x^2^_(7)_ = 6.298 and *p*-value = 0.505). For the Binary Logistic regression, a 95% confidence interval for AOR was calculated and variables with *p*-value ≤0.05 were considered as statistically associated with the development of symptoms (Symptomatic Vs Asymptomatic). An additional SPSS file supporting the analysis is attached as additional file (see Additional file 1).

## Result

### Socio–demographic, co-morbid illness and disease related variables and comparison between symptomatic vs asymptomatic

From the 778 charts, information was collected from 730 eligible charts.

The majorities of the patients involved in the study were in the two extreme age groups, < 30 years (35.1%) and ≥ 50 years (26.9%). Four hundred twenty nine (58.8%) were males. More than a quarter (28.4%) of the patients had a history of one or more pre-existing co-morbid illness. The majority had hypertension (15.2%), diabetes mellitus (11.1%), asthma (3.3%), and cardiac disease (3.4%). Twenty-one (2.9%) had a history of Khat chewing. Regarding how patient was identified as a case, 184 (25.2%) were diagnosed through contact tracing after having contact with a diagnosed person, 19 (2.6%) were diagnosed by a test done after arrival to the country from abroad, 221 (30.3%) were diagnosed during a medical visit (follow up) for other condition and the rest 303 (42.5%) were diagnosed after they presented with a complaint of symptoms of different body system.

Based on the chi-square test result, a significant difference in the presence of symptom was found among the different age groups, sex, those with a history of pre-existing co-morbid illness, cardiac disease, hypertension, diabetes mellitus and asthma.

A significantly higher proportion of patients in the age group of < 30 years were asymptomatic (66.0% Vs 34.3%, *p*-value = 0.0001). On the other hand for the rest of the three age groups, a significantly higher proportion of patients were symptomatic. The majority of females were asymptomatic (53.8% (asymptomatic) Vs 46.2% (symptomatic), *p*-value = 0.001) and males were symptomatic (40.8% (asymptomatic) Vs 59.2% (symptomatic), *p*-value = 0.001). The majority of patients with pre-existing co-morbid illness (73.8% Vs 21.7%, *p*-value = 0.0001) were symptomatic. Similarly, patients with cardiac disease, hypertension, diabetes, and asthma were symptomatic compared with those patients with no such illness (Table 1).

**Table 1:** Socio–demographic, co-morbid illness, disease related variables and comparison between Symptomatic Vs Asymptomatic patients (*n* = 730)

Variable	Asymptomatic (%) (***n*** = 366) 337	Symptomatic (%) (***n*** = 393)	Total (%) (***n*** = 730) 730	***P***-value
**Age group (in years)**				
< 30	169 (66.0)	87 (34.3)	256 (35.1)	0.0001*
30–39	74 (44.3)	93 (55.7)	167 (22.9)	
40–49	52 (44.1)	66 (55.9)	118 (16.2)	
≥ 50	42 (22.2)	147 (77.8)	189 (25.9)	
**Sex**				
Female	162 (53.8)	139 (46.2)	301 (41.2)	0.001*
Male	1730 (40.8)	254 (59.2)	429 (58.8)	
**Preexisting Co-morbid illness**				
No	292 (55.8)	231 (44.2)	523 (71.6)	0.0001*
Yes	45 (21.7)	162 (73.8)	207 (28.4)	
**Cardiac disease**				
No	337 (47.7)	369 (52.3)	704 (96.7)	0.0001*
Yes	0 (0.0)	24 (100.0)	24 (3.3)	
**Hypertension**				
No	313 (50.6)	306 (49.4)	619 (84.8)	0.0001*
Yes	24 (21.6)	87 (78.4)	111 (15.2)	
**Diabetes Mellitus**				
No	349 (52.0)	322 (48.0)	649 (88.9)	0.0001*
Yes	17 (19.3)	71 (87.7)	81 (11.1)	
**Asthma**				
No	332 (47.1)	373 (52.9)	705 (96.6)	0.004*
Yes	5 (20.0)	20 (80.0)	25 (3.4)	
**Khat chewing**				
No	332 (46.8)	377 (53.2)	709 (97.1)	0.037
Yes	5 (23.8)	16 (76.2)	21 (2.9)	

*Statistically significant

### Baseline vital sign and laboratory markers related variables and comparison between symptomatic vs asymptomatic

Based on the results of the independent t-test, symptomatic patients were relatively tachypnic (19.7/min Vs 23.2/min, *p*-value = 0.0001) and have lower oxygen saturation (95.8% Vs 93.7%, *p*-value = 0.0001). In addition, the symptomatic patients had a neutrophil predominant cell count (78.3% Vs 57.7%, *p*-value = 0.005).

A significantly raised aspartate transaminase level was seen in asymptomatic patients (77.8 IU/L Vs 44.9 IU/L, *p*-value = 0.0001) compared to symptomatic patients (Table 2).

**Table 2:** Baseline vital sign, Laboratory markers related variables and comparison between Symptomatic Vs Asymptomatic patients (*n* = 730)

Variable	Asymptomatic (Mean ± SD)	Symptomatic (Mean ± SD)	***P***-value
**Vital sign**			
Temperature (°C)	37.6 (18.1)	37.2 (16.8)	0.676
Heart rate (Beats/min)	93.8 (15.7)	97.3 (16.3)	0.187
Respiratory rate (RR/min)	19.7 (9.3)	23.2 (7.3)	0.0001*
SBP (mmHg)	132.2 (19.9)	129.9 (21.4)	0.605
DBP (mmHg)	81.9 (12.2)	79.6 (12.5)	0.631
Spo2 (%)	95.8 (1.6)	93.7 (4.5)	0.0001*
**Complete blood count**			
Hemoglobin (mg/dl)	14.8 (1.6)	15.0 (1.9)	0.764
White blood cell count (Cells/L)	7.3 (2.7)	26.2 (2.82)	0.538
Neutrophil % (%)	51.0 (24.2)	78.3 (16.8)	0.011*
Lymphocyte % (%)	24.5 (9.4)	12.0 (9.9)	0.371
**Renal function test**			
Urea (mg/dl)	23.2 (10.9)	35.7 (28.4)	0.182
Creatinine (mg/dl)	1.43 (0.8)	1.21 (0.8)	0.740
**Liver function test**			
Asparatate transaminase (IU/L)	77.8 (17.7)	44.9 (4.3)	0.0001*
Alanine transaminase (IU/L)	21.1 (13.1)	49.4 (5.1)	0.07
Alkaline phosphatase (IU/L)	74.8 (23.9)	89.0 (66.4)	0.290
**Electrolyte**			
Sodium (mEq/L)	139.8 (2.7)	142.7 (65.4)	0.599
Potassium (mEq/L)	4.6 (0.6)	4.3 (0.8)	0.489

*Statistically significant

### Factors associated with the presence of symptom in COVID-19 patients

Univariate analysis at 25% level of significance was conducted and age group, sex, hypertension, and diabetes mellitus history were found to be significantly associated with the development of symptoms. These variables were in turn included in the final multivariable regression model. Accordingly, on the multivariable binary logistic regression, age group, sex, and diabetes mellitus were found to be significantly associated with the development of symptoms at 5% level of significance. Accordingly, after adjusting for other covariates included in the final regression model, the odds of developing symptomatic disease was more likely among patients in the age range of ≥30 years compared with those patients < 30 years (AOR = 1.89, 95% CI = 1.25, 2.87, *p*-value = 0.002 for 30–39 years; AOR = 1.69, 95% CI = 1.06, 2.73, *p*-value = 0.028 for 40–49 years and AOR = 4.42, 95% CI = 2.75, 7.12, *p*-value = 0.0001 for ≥50 years).

The odds of developing symptomatic COVID-19 among males was 1.76 times than female patients (AOR = 1.76, 95% CI = 1.26, 2.45, *p*-value = 0.001).

Regarding the history of pre-existing co-morbid illness, the odds of developing symptomatic disease among diabetic patients was 3.90 times compared to patients with no such illness (AOR = 3.90, 95% CI = 1.92, 7.94, *p*-value = 0.0001) (Table 3).

**Table 3:** Results for the final multivariable binary logistic regression model among COVID-19 patients (*n* = 730)

Variable	COR (95% CI)	AOR (95% CI)	***P***-value
**Age group (in years)**			
< 30	1	1	0.0001*
30–39	2.44 (1.64, 3.64)	**1.89 (1.25, 2.87)**	0.002*
40–49	2.47 (1.58, 3.85)	**1.69 (1.06, 2.73)**	0.028*
≥ 50	6.79 (4.43, 10.45)	**4.42 (2.75, 7.12)**	0.0001*
**Sex**			
Female	1	1	0.001*
Male	1.69 (1.26, 2.28)	**1.76 (1.26, 2.45)**	
**Hypertension**			
No	1	1	0.05
Yes	3.71 (2.29, 5.98)	1.73 (0.99, 3.01)	
**Diabetes Mellitus**			
No	1	1	0.0001*
Yes	7.21 (3.65, 14.23)	**3.90 (1.92, 7.94)**	

*COR* Crude Odds ratio, *AOR* Adjusted Odds ratio, *CI* Confidence interval

*Statistically significant

## Discussion

This study has assessed factors affecting the developing symptomatic COVID-19 disease among RT-PCR confirmed patients admitted at MCCC. The chi-square test result of the study shows that a significant difference in having symptomatic Vs asymptomatic infection is observed in some of the variables showing that symptomatic disease is significantly associated with old age, male sex, and having one or more co-morbid illness. Based on the t-test, symptomatic infection is significantly associated with a relatively higher breathing rate, a lower than normal level of oxygen in the blood, neutrophil predominance, and lower aspartate transaminase. The derangement in laboratory biomarkers could be attributed to the infection itself or due to the medication taken for other medical conditions or treatments taken to treat the consequences of the infection before admission.

On the multivariable binary logistic regression; age group, sex, and diabetes mellitus were significantly associated with the development of symptoms.

Older age was found to be a significant factor that affects the development of symptomatic COVID-19. Accordingly, after adjusting for other covariates, the odds of presenting with symptomspresenting with symptoms were more likely among patients in the age range of 30 years and above compared with those patients < 30 years old. The odds are higher (more than four times) for those patients 50 years and above. That means older age groups are more susceptible to developing symptomatic disease once they are infected. This could be because, as it is already known, the body’s immune defense mechanism tends to get weaker with age making aged people more susceptible to severe disease and worse outcomes from both infectious and non-infectious diseases. This is especially compounded by the presence of other underlying conditions (comorbidities) which are highly likely to be developed as someone gets older. More than a quarter (28.4%) of the participants included in the study have also a history of one or more preexisting co-morbid illness.

Male sex was associated with symptomatic COVID-19 infection. The odd of having symptomatic COVID-19 among males was 1.76 times than female patients. This disparity in sex in terms of symptoms and also acquiring the disease, having a more severe disease course, and worse outcome is observed in many countries with reports showing that a significantly higher proportion of men are getting hospitalized and dying from the disease compared to females. Different explanations have been given to the disparity, from genetics up to behavioral factors as contributors. Studies show that Angiotensin-converting enzyme 2 (ACE2) which is found to be a receptor for the SARS-CoV-2 is found to be present in high concentration among males, which makes them vulnerable to have a more severe disease pattern and worse outcome [9–15]. It is also hypothesized that men might not be as good as women in terms of taking necessary precautions to prevent acquiring the disease and also in seeking early medical attention if they get infected and become symptomatic. Understanding these modifiable behavioral factors is important to provide intervention to decrease severe disease and worse outcomes in men.

The other important factor that affected the development of symptomatic infection was being a diabetic patient. The odds of developing symptomatic disease among diabetic patients were 3.90 times compared to patients with no such illness. It is well known that diabetes mellitus is one of the chronic medical illnesses that is associated with depressed body immunity that will leave the patient to be susceptible to any infectious diseases. It also increases the patients’ probability of developing another comorbid illness which in turn could result in diabetes disease progression and development of short and long-term complications including poor glycaemic control that will make the patient more susceptible to external invaders resulting in symptomatic infection, severe disease, and poor outcome. This is also found to be the case in other studies, where having diabetes is associated with unfavorable clinical presentation and COVID-19 outcome [17–20].

The findings of the study have to be interpreted with the following strength and limitation in mind. Its strength is that the study was conducted in the most representative COVID-19 Care center in the country with the largest bed number, highest patient flow and admission of all types of patients including asymptomatics from all around the country especially at the beginning of the pandemic, the time when the study was conducted. Its limitation was important potential risk factor variables like BMI, additional laboratory and radiologic data were not consistently available for all patients so could not be considered in the final regression model.

## Conclusion

Developing a symptomatic COVID-19 infection was found to be affected by the following factors; old age, male sex, and being diabetic.

Therefore, patients with the above factors should be given enough attention, including inpatient management, to pick symptoms earlier and to manage accordingly so that these patients can have a favorable treatment outcome. In addition, further study should be conducted to assess the role of behavioral factors as a cause of the disease course disparity between the two sexes, so that intervention can be made on the identified modifiable behaviors.

## Supplementary Information

**Additional file 1.** Asymptomatic Vs Symptomatic COVID-19. Data used for analysis.

## Data Availability

All relevant data are available upon reasonable request.

## Abbreviations

CI: Confidence Interval
COVID-19: Coronavirus Disease 2019
OR: Odds Ratio
SARS-COV-2: Severe Acute Respiratory Syndrome Coronavirus 2
RT-PCR: Real Time Polymerase Chain Reaction
